# Effect of exercise-induced muscle fatigue on reaction times under postural perturbation conditions in individuals with and without chronic low back pain

**DOI:** 10.3389/fspor.2026.1883709

**Published:** 2026-08-31

**Authors:** Hao Xie, Wenwu Xiao, Jiahui Peng, Huaichun Yang, Haoyu Xie, Zengming Hao, Zifeng Li, Haian Mao, Chuhuai Wang

**Affiliations:** 1 Department of Rehabilitation Medicine, The First Affiliated Hospital of Sun Yat-sen University, Guangzhou, Guangdong, China; 2 China Three Gorges University, Department of Rehabilitation Medicine, Affiliated Renhe Hospital, Yichang, Hubei, China; 3 Yichang Key Laboratory of Brain Neuroregulation, Yichang, Hubei, China; 4 Department of Rehabilitation Medicine, The Affiliated Suzhou Hospital of Nanjing Medical University, Suzhou Municipal Hospital, Gusu School, Nanjing Medical University, Suzhou, Jiangsu, China; 5 Department of Traditional Chinese Medicine, The First College of Clinical Medical Science, China Three Gorges University/Yichang Central People’s Hospital, Yichang, Hubei, China

**Keywords:** chronic low back pain, muscle fatigue, postural control, reaction time, surface electromyography

## Abstract

**Background:**

Total reaction time (TRT), composed of premotor time (PMT) and electromechanical delay (EMD), is susceptible to muscle fatigue and can be affected by pain. Limited evidence is available regarding the postural control capacity of patients with chronic low back pain(cLBP) following muscle fatigue-induced postural perturbation. This study aimed to examine the impact of pain and muscle fatigue on reaction time, so as to gain insights into the neuromuscular control strategy changes associated with muscle fatigue effect of cLBP.

**Methods:**

Twenty-four subjects with cLBP (cLBP group) and twenty-two healthy individuals (HC group) were tested by Biering-Sorensen Test to generate exhaustive muscle fatigue. TRT, PMT and EMD were recorded by surface electromyography during the arm raising task with visual cues prior to and following muscle fatigue. The mean difference (MD) of reaction time was calculated before and after muscle fatigue, denoted as MD_TRT_, MD_PMT_, and MD_EMD_, respectively. Besides, the fear avoidance beliefs questionnaire (FABQ) and visual analogue scale (VAS) was evaluated before muscle fatigue in cLBP group.

**Results:**

The TRT and PMT were significantly prolonged after muscle fatigue in the cLBP group compared with before muscle fatigue (Z = −3.371, *P* *<* 0.001; Z = −3.286, *P* *<* 0.001, respectively). Meanwhile, the cLBP group had significantly shorter TRT and PMT before muscle fatigue than HC group (Z = −3.299, *P* *<* 0.001; Z = −3.256, *P* *<* 0.001, respectively). Additionally, the correlation analysis manifested that MD_PMT_ and MD_TRT_ were positively correlated with FABQ (r = 0.422, *P* *=* 0.040; r = 0.418, *P* *=* 0.042) and VAS (r = 0.546, *P* *=* 0.006; r = 0.564, *P* *=* 0.004) separately.

**Conclusions:**

The reaction time would be altered by chronic pain in neuromuscular control processes, and muscle fatigue could further induce it delay. Besides, this reaction time delay was positively correlated with pain level and fear-avoidance beliefs. These findings highlight the importance of the muscle fatigue effects with cLBP, such as avoiding exhaustive muscle fatigue and paying more attention to fear-beliefs and pain during rehabilitation.

**Trial registration:**

This trial is registered at [chictr.org.cn], number [ChiCTR2300074348]. Registered August 4, 2023.

## Background

Chronic low back pain (cLBP) is one of the leading causes of years lived with disability, affecting individuals across of all ages and imposing a substantial disease burden on global healthcare system (1). Non-specific chronic low back pain(NCLBP) accounting for approximately 90% of all cases (2, 3). However, the pathogenesis of this category population remains unclear, and the current treatment options often yield unsatisfactory outcomes (4). Therefore, it deserves to further explore the underlying mechanisms and associated risk factors of NCLBP.

Exercise-induced muscle fatigue is a common phenomenon in daily life, which refers to the inability of muscles to maintain the expected strength and specific level of function during any physical activity (5). Exercise-induced muscle fatigue can impact the neuromuscular control of the body (6), leading to changes such as altered proprioceptive sensitivity, weakened muscle contraction function, decreased postural stability. These changes may contribute to the occurrence of chronic pain (7). For instance, one study has demonstrated that inducing isolated fatigue in the back and hip joints can reduce sagittal postural stability in individuals with NCLBP, as opposed to healthy individuals. It also suggested that muscle fatigue is able to cause the recurrence of low back pain (8). Further, studies have indicated that muscle fatigue is a common phenomenon in cLBP patients, with an incidence ranging from approximately 26 to 69.7% (9, 10). The study by Saravanan et al. demonstrates that in adult patients with cLBP, fatigue is associated with heightened depressive symptoms, increased pain burden, and greater functional impairment (11). Hence, investigating the role of muscle fatigue may contribute to a deeper understanding of cLBP.

The neuromuscular response process could be altered under conditions of fatigue (12), and prolonging it can decrease the postural control ability as well as increase the risk of physical injury (13). Total reaction time (TRT), which measures the time taken to respond to various stimulus disturbances, is considered a reliable indicator of the velocity and efficiency of the neuromuscular response process (5). TRT is comprised of two components: premotor time (PMT) and electromechanical delay (EMD). PMT represents the time interval from stimulus onset to muscle electrical signal generation and may be correlated with pain (14) as well as fear beliefs (15). It has been suggested that PMT might have cognitive and psychological components (5, 16). EMD is the time from muscle electrical signal excitation to the onset of muscle mechanical contraction, representing the process from myoelectric activation to muscle force production. The length of EMD is closely related to exercise training (17), muscle activity and metabolism (18, 19). Studying these phases separately can help clarify the specific process of neuromuscular reaction in response to the various stimuli of different circumstances, which is applicable to muscle fatigue and chronic pain research. For instance, a previous study demonstrated that basketball players with cLBP exhibited significantly longer PMT and TRT in lower limb muscles following fatigue during a choice reaction time task, compared with healthy controls to predict the risk of lower limb injury (20). Another study examining the effects of fatigue on patellar tendon reflex responses in males and females also found that the TRT(especially in EMD period) was significantly increased in females after fatigue but not in males (21). These studies suggest that reaction time is not only affected by muscle fatigue but also by the presence of chronic pain. Nevertheless, the muscle fatigue effects on reaction time in cLBP under postural perturbation conditions has seldomly reported. The change in fear-avoidance beliefs can significantly affect the motor function of patients with cLBP (22). Research has identified fear-avoidance beliefs (the conviction that physical activity exacerbates or delays recovery from low back pain) as a key psychological contributor to the development and persistence of low back pain, and moderate to high levels of fear-avoidance beliefs are significantly associated with both greater pain intensity and longer pain duration (23). But it remains to be determined whether muscle fatigue–induced alterations in neuromuscular reaction time are associated with pain perception and fear-related cognitive beliefs.

As noted previously, we propose the scientific hypothesis that individuals with cLBP would experience reaction time change due to muscle fatigue when faced with postural perturbations. In this research, surface electromyography (s-EMG) was used to measure the reaction time before and after exercise-induced muscle fatigue under postural perturbation condition, aiming to further perform a component-level analysis of TRT, PMT, and EMD under these conditions and assess whether the observed changes were correlated with pain-related fear scores. Notably, the cLBP data in the current study are derived from an extension of our previously published work that examined cLBP participants only (24). The present study adds a healthy control group and investigates new research questions related to between-group differences for how muscle fatigue affects neuromuscular control strategies.

## Methods

### Study design and setting

This analytical observational study was approved and supervised by the Ethics Committee of the First Affiliated Hospital, Sun Yat-sen University [Ethics Approval No. (2023) 386-1], registered in the China Clinical Trial Registry Center (No. ChiCTR2300074348), and conducted by the Department of Rehabilitation Medicine. The Declaration of Helsinki and all human experimentation rules were respected (25). Subjects were informed of the potential risks (e.g., electrode pad allergy, muscle soreness) and their consents were obtained before the trial. In order to maintain the quality of the report, we followed the STROBE standards and checklist (26).

### Participants

Subjects were recruited through advertisements and screened by a professional physician. The inclusion criteria of cLBP group were as follows: 1) in accordance with the medical diagnoses for NCLBP (27); 2) age between 18 and 59 years; 3) pain was located between the 12th rib and the gluteal sulcus, the symptom lasted more than 3 months, and with one episode of recurrent low back pain in the past 3–12 months; 4) the VAS score were greater than 3/10 points; 5) Right-dominant hand; 6) subjects in healthy group were matched with cLBP group for general epidemiologic characteristics such as age, gender, height, weight, education level, and body mass index.

Exclusion criteria were: 1) specific low back pain (e.g., spinal tumor, tuberculosis, fracture, spinal stenosis, intervertebral disc herniation.); 2) history of spinal or pelvic surgery within the past two years; 3) undergoing low back pain physical rehabilitation or oral medication; 4) body mass index above 30 kg/m^2^; 5) pregnant or preparing for pregnancy. 6) unable to cooperate with the examination on account of serious cognitive psychological or with audiovisual disorders.

### Sample size calculation

In the preliminary study, we applied the TRT parameter changes (before vs. after muscle fatigue) of eight subjects, and compared the results between cLBP (0.1642 ± 0.0835) and HC (0.1049 ± 0.0485). These data were then used to conduct the sample calculation by G-Power (version 3.1.9.4). The Cohen's d value was 0.868, using two-sided test with *α*= 0.05, power(1-*β*) = 0.8, and allocation ratio(N1/N2) = 1. The sample total size was calculated to be 44, and 22 in each group.

### Instruments and measures

In this trial, s-EMG was employed to gauge reaction time under dynamic postural perturbation conditions before and after muscle fatigue. The visual analogue scale (VAS) and fear-avoidance beliefs questionnaire (FABQ) were used to assess pain perception and fear-avoidance beliefs in cLBP.

#### Biering-Sorensen test

**Figure 1:**
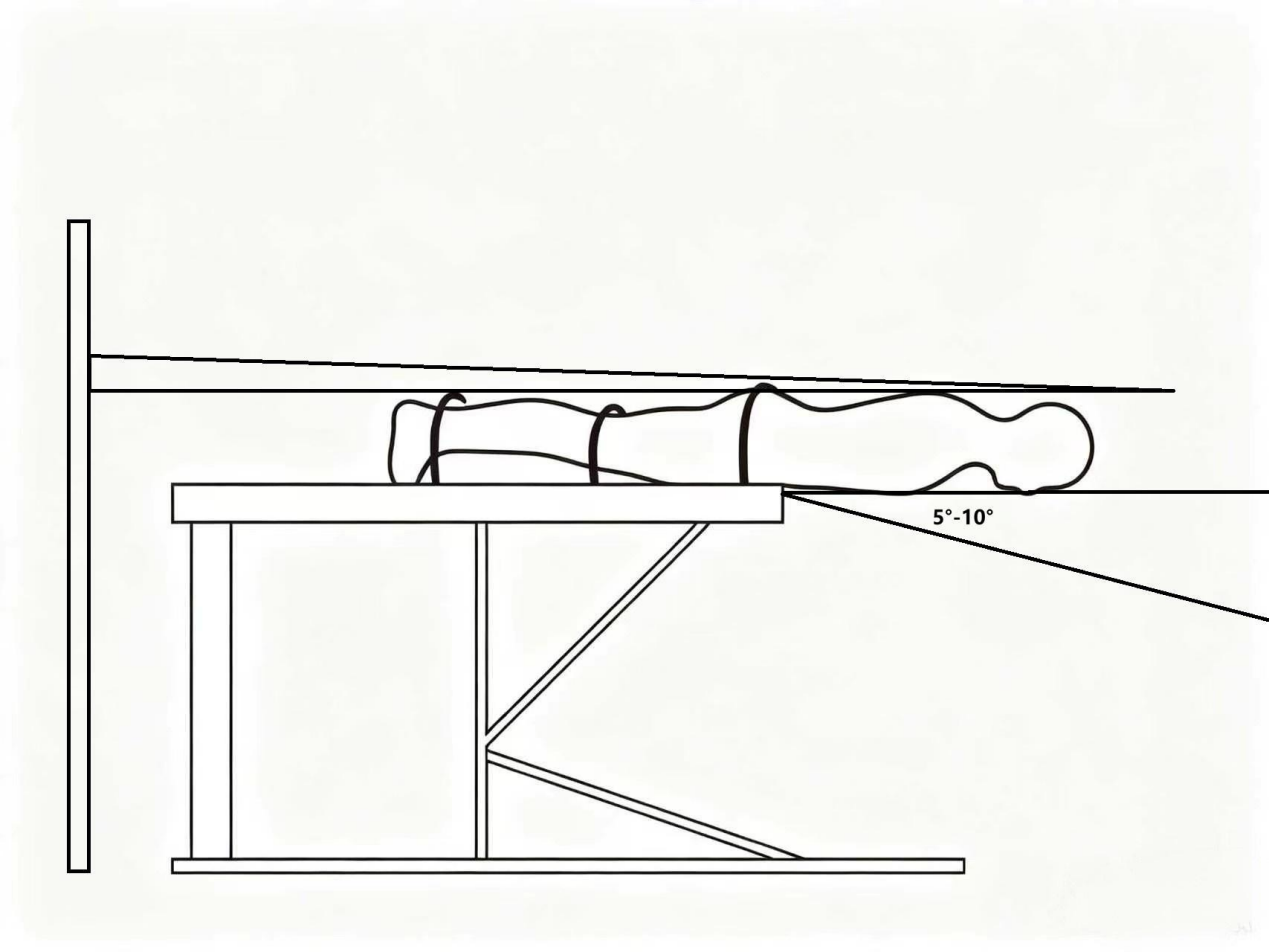
The schematic diagram of the muscle fatigue test (biering-sorenson test, BST).

The Biering-Sorensen Test (BST) is a well-established and commonly used paradigm to induce muscle fatigue. It is considered reliable, valid, and safe, and has been used in numerous studies involving individuals with low back pain (28). In this study, we used the BST to induce muscle fatigue in all subjects. The details were as follows: as presented in Figure 1, the subject lay prone on a treatment bed, and the parts below the iliac spine were fastened with belt at the hip, popliteal fossa, and ankle level. In the meantime, the parts above the iliac spine were suspended outside the treatment bed. We set up a horizontal bar device to indicate the horizontal position of the subject's trunk, and a visual angle indication device to assist the experimenter in judging the subject's trunk position. Once the test started, subjects were instructed to cross over upper limbs to the chest and keep their body in a horizontal line. During the BST hold, subjects were verbally reminded by the experimenter at regular intervals to avoid any compensatory movements (e.g., hip hyperextension, pelvic anterior tilt, trunk rotation) that could assist in maintaining the horizontal trunk posture. The test was ended when subjects were too exhausted to maintain horizontal position(deviation exceeding 5°–10° from the visual angle indicator or sustained displacement from the back support bar for more than 10s) or experienced pain (29, 30). The duration time of BST was recorded. Immediately upon termination, the investigator assisted the participant to transition from prone to standing and guided them to the testing platform. The sEMG electrodes were rapidly checked for connectivity, without any re-preparation. Rapid dynamic postural perturbation test was performed within 20 s (31).

#### Dynamic postural perturbations test

**Figure 2:**
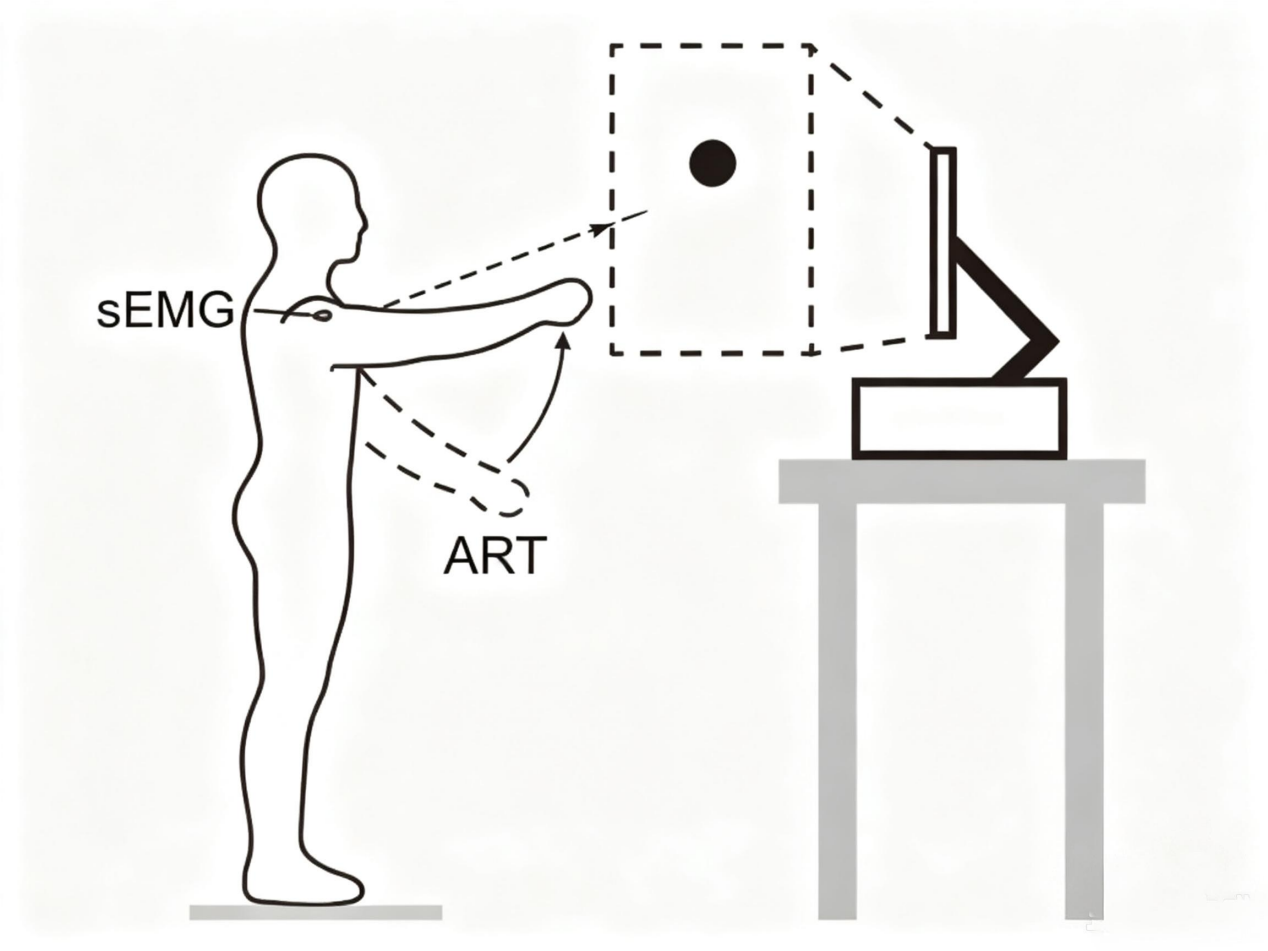
The schematic diagram of arm-raising test (ART).

The rapid arm-raising test (ART) was executed to detect dynamic postural perturbation in all subjects both before and immediately after muscle fatigue. The ART is a reliable internal dynamic postural perturbation paradigm, and we slightly modified it referring to previous research (32). The detailed process of ART unfolded as follows: subjects stood barefoot on a platform with their hands naturally hanging down, and palms lightly touching the lateral thighs. Trunk was instructed to be upright with neutral head position. A visual arm-raising signal (depicted as a solid black circle lasting for a duration of 3 s), compiled by Psychopy software (version 2022.1.3), was presented on the monitor which was placed 2 meters in front of subjects at the eye level. In this procedure, the signal was presented three times in 30 s randomly. The visual stimulus was presented for 3 s, and the inter-stimulus interval was randomized between 8 and 12 s (uniform distribution, mean 10 s) to prevent temporal anticipation. Upon signal onset, participants were instructed to rapidly elevate their right arm to the horizontal plane (90°of shoulder flexion confirmed by a pre- placed marker). Once the visual signal appeared, the subject was instructed to raise the right arm to the shoulder plane with maximal voluntary acceleration, and to return to the neutral position when the signal disappeared. In order to ensure subjects were familiar with the test, they were given two or three opportunities to practice the procedure before the data collection. Electromyographic signals were recorded during three trials of rapid arm extension in the formal test. A schematic diagram of ART is shown in Figure 2.

#### Reaction time test

**Figure 3:**
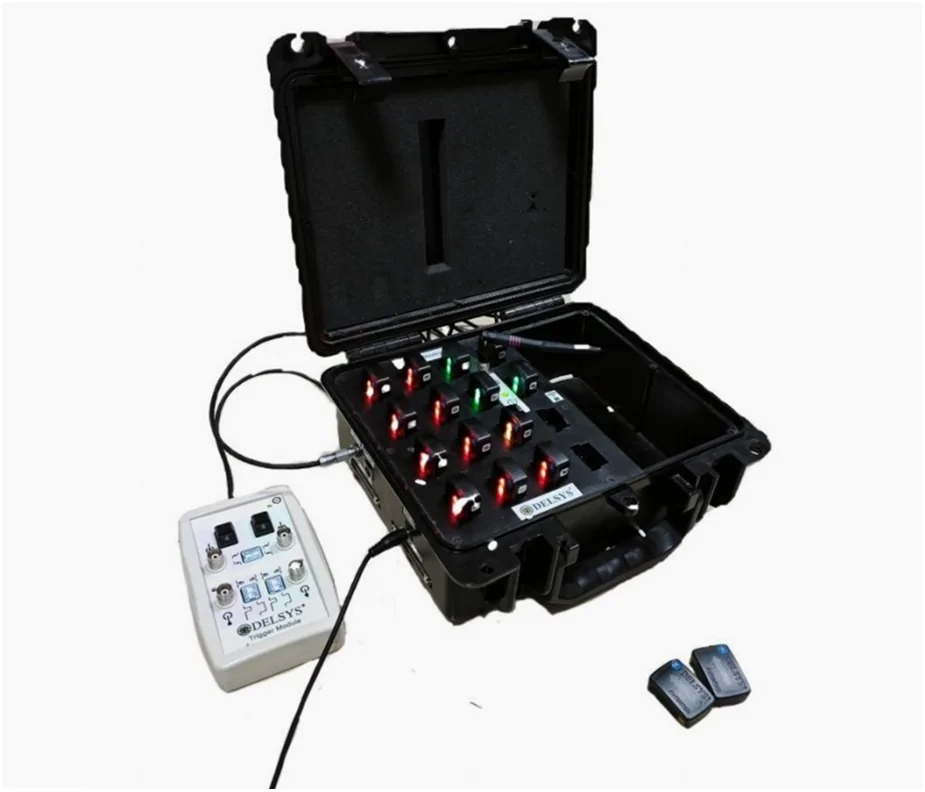
s-EMG device (trigno™, delsys incorporated, USA) and trigno Avanti™ sensor.

The wireless s-EMG technology (Trigno™, Delsys Incorporated, USA), as shown in Figure 3, represents a prevalent method to detect neuromuscular reaction time, and we used it to record PMT, EMD and TRT, separately (5, 33). Through technical modification in this study, the physical impulses emitted from Psychopy software would simultaneously trigger the acquisition of the s-EMG, which realized the absolute synchronous recording between ART signals and s-EMG acquisition. Before starting s-EMG recording, s-EMG signals from each channel were processed using a fourth-order Butterworth band-pass filter (20–450 Hz) to retain physiologically relevant frequency components, followed by notch filtering at 50 Hz to suppress power-line interference (34), and all signals were acquired at a sampling frequency of 2000Hz (35). EMG onset criteria was defined as the time point at which the rectified EMG amplitude exceeded the mean baseline activity (recorded for 100 ms prior to the expected onset) by 3 standard deviations for a continuous period of at least 25 ms (36). Onset times were identified automatically and subsequently visually inspected and verified by two independent examiners. Trials with ambiguous onsets were excluded from analysis. To ensure that the analysis is based on purely unconscious reflexive responses rather than the subjects’ voluntary or pre-programmed actions, we set a hard exclusion window of 70 ms after the disturbance - any trial in which a clear electromyographic signal appeared within this window was regarded as a predictive response and was excluded (37). The specific process was as follows: the s-EMG electrodes model were Trigno Avanti™ Sensor, placed on the mid- belly of the right anterior deltoid at the middle one-third of the line from the acromion to the deltoid tuberosity, with electrodes aligned longitudinally along the muscle fibers (before placing the electrodes, the skin keratin and dirt was removed by scrub cream, and then cleaned with 75% alcohol to reduce the impedance). All electrode placements were performed by a single trained researcher to reduce inter- operator variability. The ART onset (T_0_) was defined as the instant the visual ‘a solid black circle’ signal appeared on the screen. The onset time of the initial mechanical contraction moment was recorded through displacement sensor in s-EMG, which was denoted as T_torque_. Trigno Avanti™ Sensor have a built-in 9 DOF inertial measurement unit which can relay acceleration, rotation and earth magnetic field(compass) information. Meanwhile, the time point of the myoelectrical activation was characterized as T_electrical_. TRT, calculated as the difference between T_torque_ - T_0,_ represented the time interval from the appearance of ART signal to the mechanical contraction moment. PMT was determined by the computation T_electrical_ - T_0_, referring to the time interval from the appearance of ART signal to the myoelectrical activation moment. EMD was the time interval from the myoelectrical activation to mechanical contraction moment, which was computed through the equation T_torque_ - T_electrical_. Besides, we calculated the mean difference (MD) change of TRT, PMT and EMD before and after muscle fatigue, which were denoted as MD_TRT_, MD_PMT_ and MD_EMD_, respectively.

#### Pain and fear-avoidance belief assessment

VAS and FABQ demonstrate a good reliability and validity to assess the degree of pain and fear- avoidance beliefs on daily activities and work in cLBP (38, 39). The VAS scores range from 0 to 10, with higher scores indicating more severe pain. Specifically, the scale encompasses categories such as: absence of pain (0), mild pain (1∼3), moderate pain (4∼6), and severe pain (7∼10). The FABQ consists of 16 items, in which 5 items assess the fear avoidance beliefs related to the impact of physical activity on chronic pain, the other 11 items refer to work-related activities. The total score of FABQ is 96, each item ranging from 0 to 6 points. Elevated scores on FABQ represent a higher degree of fear-avoidance beliefs. In this trial, we obtained VAS and FABQ scores through electronic questionnaire in cLBP group before muscle fatigue. The cLBP data set builds upon our prior study (24), while the current work introduces a newly recruited healthy control group.

### Bias

To ensure the mitigation of measurement errors and result biases, meticulous quality control measures were implemented, encompassing the following aspects: 1) a comprehensive explanation of the assessment procedure was provided to enhance collaboration among participants and researchers; 2) One examiner performed participant grouping, while a second examiner—completely blinded to group allocation—administered BST for the total endurance time and recorded ART-mode EMG signals, all under double-blinded conditions. 3) the average reaction time of three ART measurements was included for final data analysis. 4) additionally, the ART signal was fully synchronized with the s-EMG acquisition through meticulous technique refinement, which effectively controlled the acquisition bias of reaction time.

### Data processing and statistical analysis

We employed Matlab R2022a software (MathWorks, Natick, MA, USA) to process and analyze the s-EMG signals. Subsequent statistical analysis was performed by SPSS 20.0 (IBM, Chicago, USA). Normality test was processed first for all continuous variables (age, height, weight, BMI, TRT, PMT, EMD, VAS, FABQ, BST-TET). The result was expressed as mean ± standard deviation (MD ± SD) when the data adhered to a normal distribution. The paired t-test was used for comparisons within groups, and the independent t-test for comparisons between groups. In cases where the data deviated from a normal curve, the results were represented by median (25%, 75% interquartile values) and non-parametric tests were used for inter- and intra-group comparisons. Pearson or Spearman was used to analyze the correlation of MD_TRT_, MD_PMT_, and MD_EMD_ with the VAS and FABQ separately, based on normality test results of this data. *p* < 0.05 indicates a statistical significance.

## Results

### Demographic characteristics and research flowchart

The present study initially enrolled a total of 48 participants, including 23 healthy individuals (HC group) and 25 cLBP subjects (cLBP group). One healthy participant withdrew from the trial for personal reasons, and one participant from the original cLBP cohort was excluded due to excessive EMG artifacts that compromised the reliability of motor unit action potential decomposition, yielding a final analytical sample of 46 participants.

**Figure 4:**
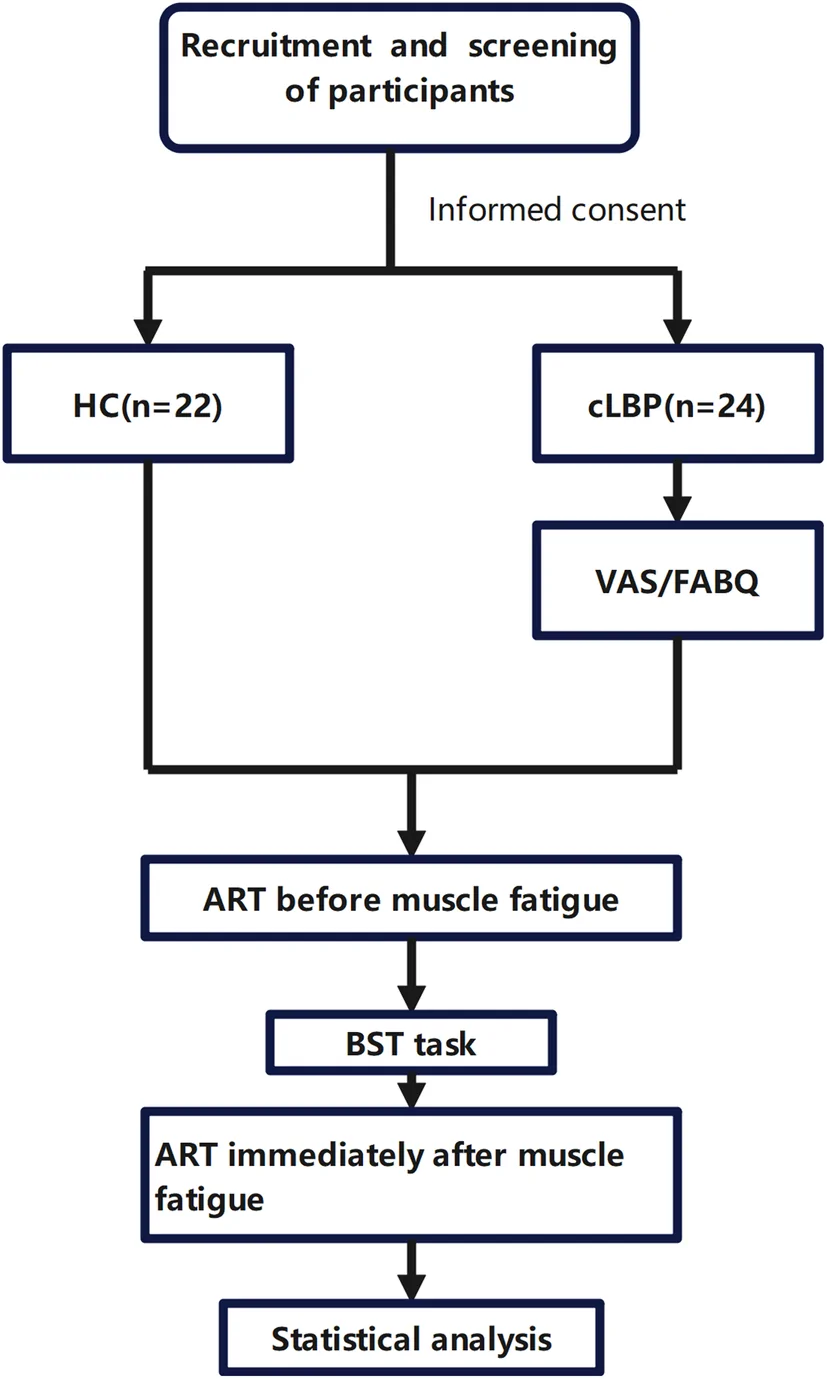
the research flowchart.

As shown in Table 1, no significant differences were observed in baseline characteristics between the two groups, such as gender, age, weight, BMI, height, education level. In addition, The VAS, FABQ scores and BST-TET of cLBP group were also exhibited in Table 1. The experimental procedure is illustrated in Figure 4.

**Table 1:** Demographic variables of the subject's data.

		HC(*n* = 22)	cLBP (*n* = 24)	**t/** *χ* ^2^ **/Z**	*p*
Gender (male/female)^a^		10/12	10/14	0.05	1
Age (years)^c^		29.35 (23.00, 33.00)	31.56（25.00, 36.00）	−1.20	0.23
Weight (kg)^b^		62.20 ± 11.76	58.72 ± 10.29	1.09	0.28
Height (m^b^		1.64 ± 0.07	1.66 ± 0.08	−0.83	0.41
BMI (kg/m^2^)^b^		22.98 ± 3.73	21.17 ± 2.55	1.97	0.06
Education level ^c^	Junior college or less	1	3	−1.59	0.11
	undergraduate	5	8		
	Postgraduate or higher	16	13		
BST-TET (s)^b^		100.13 ± 34.95	105.08 ± 36.83	−0.48	0.64
VAS			5.28 ± 1.62		
FABQ (score)^b^			47.33 ± 18.43		

a Value is presented as sex composition ratio.

b Values are presented as mean ± standard deviation.

c Value is presented as median (25%∼75% interquartile range).

BST-TET, the total endurance time of Biering-Sorensen Test; VAS, visual analogue scale; FABQ, Fear Avoidance Beliefs Questionnaire.

### The reaction time change before and after exercise-induced muscle fatigue

**Figure 5:**
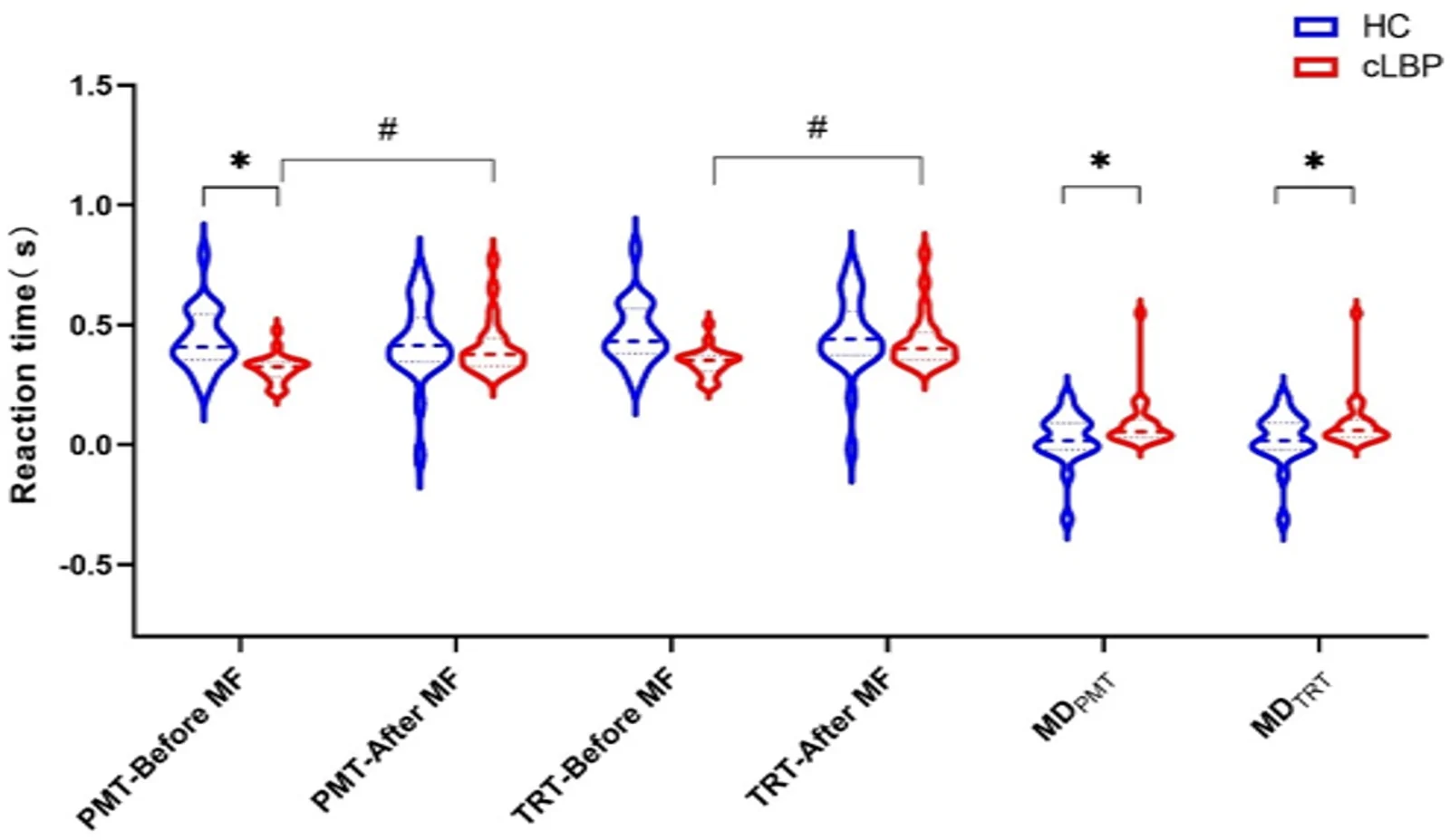
Comparison of PMT and TRT between two groups. PMT, TRT, MD_PMT_ and MD_TRT_ before and after muscle fatigue were represented by median (25%, 75% interquartile values). * *P* < 0.05, inter-group comparison (non-parametric test); # *P* < 0.05, intra-group comparison (non-parametric test).

**Figure 6:**
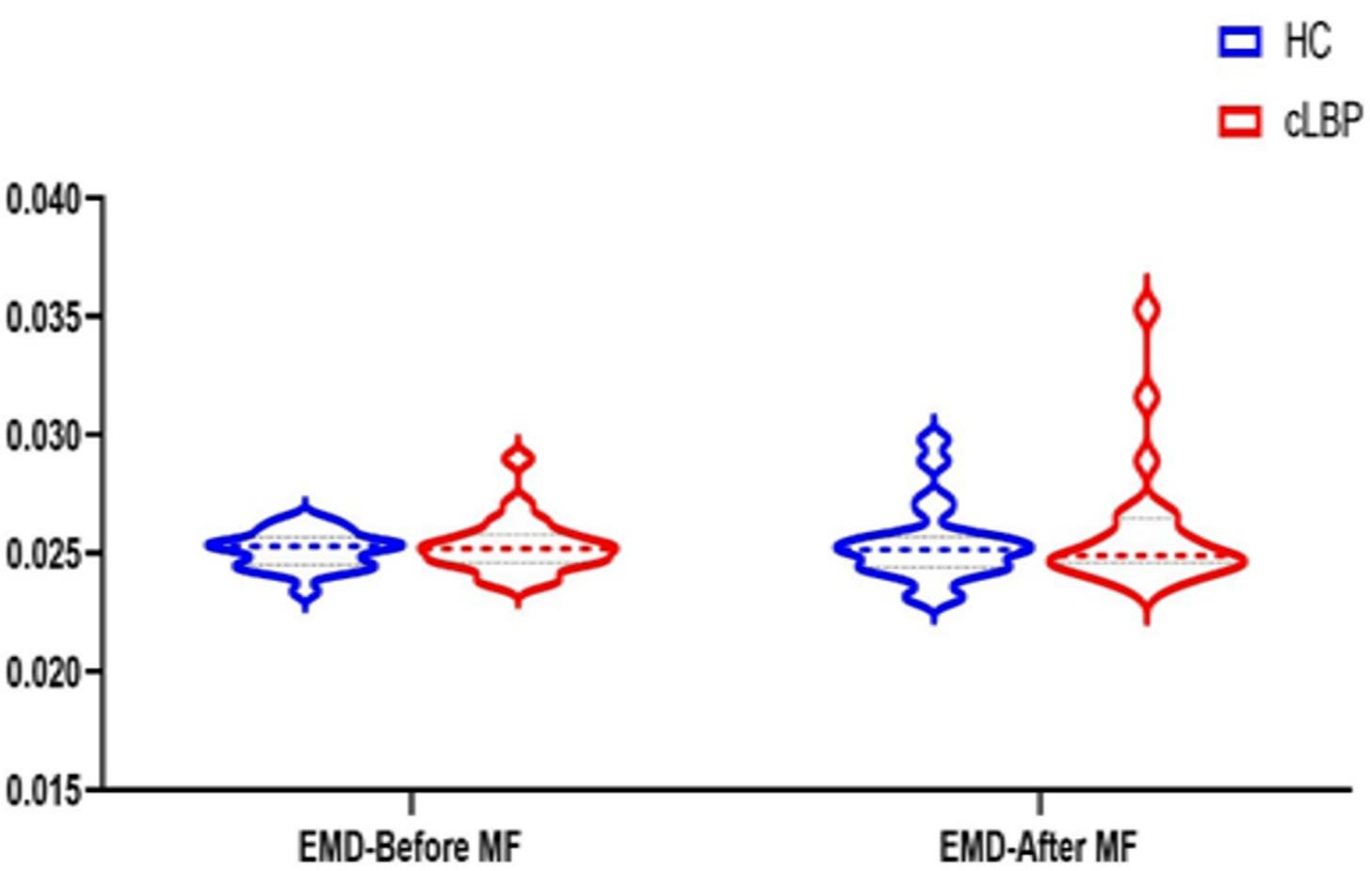
Comparison of EMD between two groups. EMD before and after muscle fatigue was represented by median (25%, 75% interquartile values).

Before inducing muscle fatigue, both TRT and PMT were significantly decreased in cLBP group compared to HC group (Z = −3.299, *P* *<* 0.001; Z = −3.256, *P* *<* 0.001, respectively). Furthermore, following muscle fatigue induction, both TRT and PMT were significantly prolonged in cLBP group (Z = −3.371, *P* *<* 0.001; Z = −3.286, *P* *<* 0.001, respectively). However, no statistical differences were observed in EMD within the cLBP group, nor in TRT, PMT, and EMD within the HC group. To assess the change in magnitude of reaction time change parameters before and after induced muscle fatigue in the cLBP group, we calculated the mean difference (MD) of TRT and PMT (referred to as MD_TRT_ and MD_PMT_ respectively). Furthermore, analysis of reaction time changes before and after fatigue induction revealed that both the mean dual-task performance reaction time MD_PMT_ and the mean dual-task response time MD_TRT_ were significantly prolonged in the cLBP group compared with the healthy control (HC) group, with statistically significant between-group differences (Z = −2.066, *p* = 0.039; Z = −2.044, *p* = 0.041). Nevertheless, no statistical differences were observed neither in EMD before muscle fatigue, nor in TRT, PMT, and EMD after muscle fatigue. The details were presented in Figures 5, 6.

### Correlation analysis between the mean difference (MD) of reaction time and VAS, FABQ

**Figure 7:**
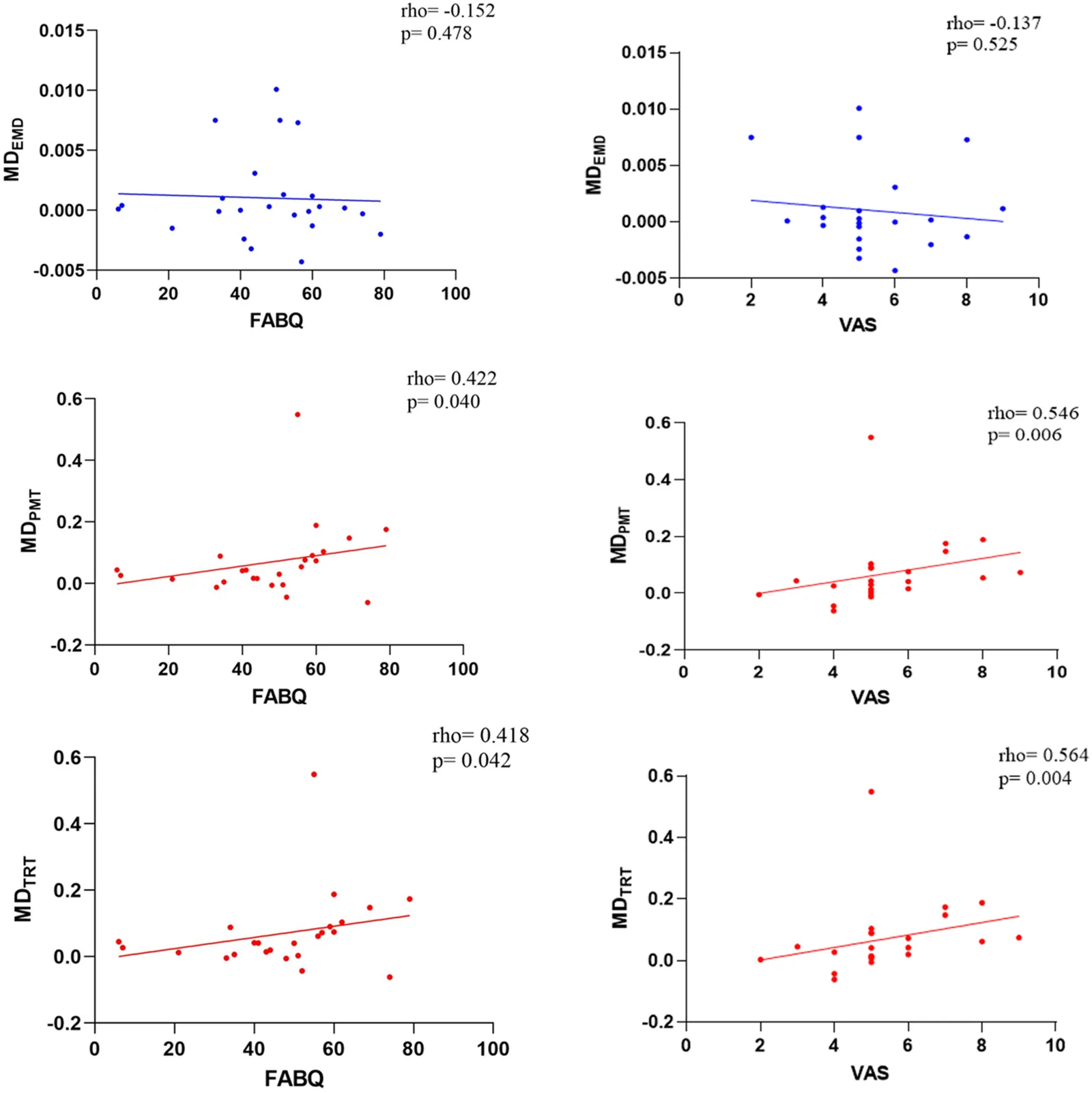
Correlation analysis of MD_TRT_, MD_PMT_, MD_EMD_ with VAS or FABQ. The blue scatter plot shows no statistically significant linear correlation, whereas the red scatter plot demonstrates a strong and statistically significant positive linear correlation, as assessed by Spearman's correlation coefficient.

The details were exhibited in Figure 7. Correlation analysis revealed that MD_TRT_ and MD_PMT_ manifested positive correlations with FABQ (r = 0.418, *P* *=* 0.042; r = 0.422, *P* *=* 0.040, respectively), and similarly exhibited positive correlations with VAS (r = 0.546, *P* *=* 0.006; r = 0.564, *P* *=* 0.004, respectively).

### Correlation analysis between BST-TET and VAS, FABQ

**Figure 8:**
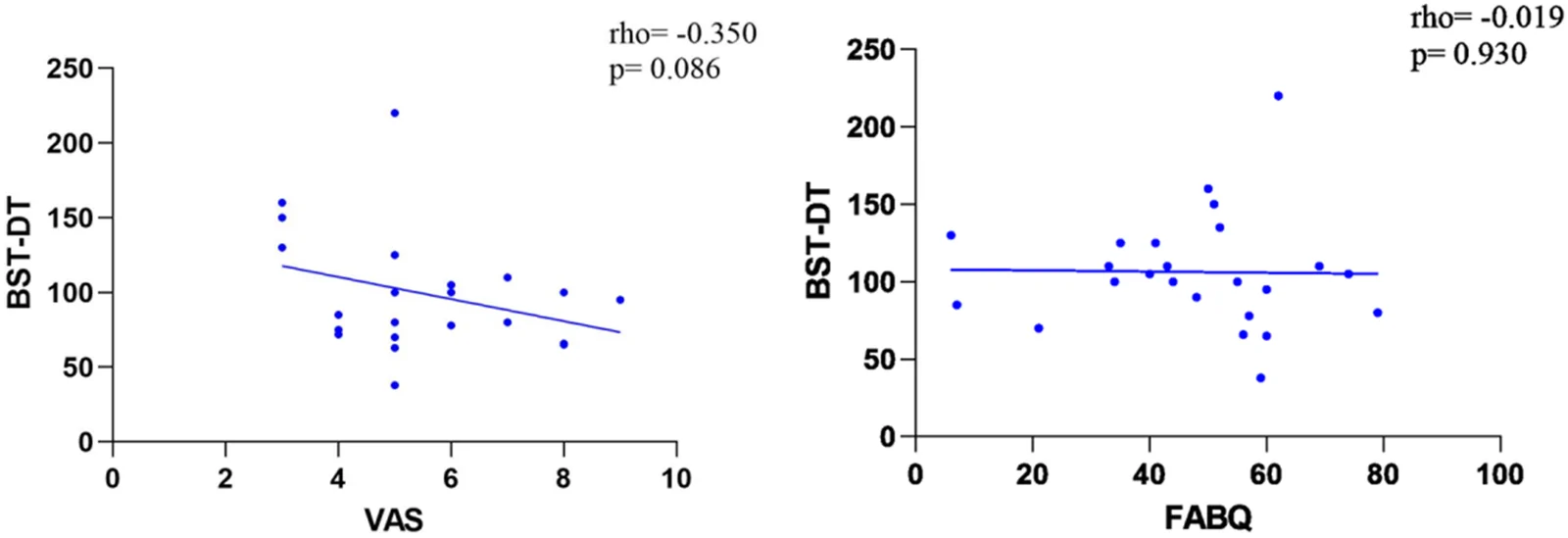
Correlation analysis between BST-TET and VAS, FABQ. The blue scatter plot shows no statistically significant linear correlation, as assessed by Spearman's correlation coefficient.

As demonstrated in Figure 8, the correlation analysis results showed a lack of significant correlation between BST-TET and FABQ (r = −0.019, *P* *=* 0.930) as well as between BST-TET and VAS (r = −0.350, *P* *=* 0.086).

## Discussion

This study carried out the BST to induce muscle fatigue and performed an ART in response to sudden visual stimulation in people with and without cLBP, and determined the reaction time before and after fatigue. We found that: 1) the TRT and PMT before muscle fatigue were reduced in cLBP group, compared to HC group (*P* *<* *0.05*); 2) following muscle fatigue, the TRT and PMT increased in the cLBP group (*P* < 0.05), and 3) the degree of MD_TRT_ and MD_PMT_ were positively correlated with both pain perception and fear-avoidance beliefs.

### Chronic pain and reaction time

Reaction time is usually measured by limb movement response to target stimulation (40), reflecting an individual's ability to cope with the danger on sudden perturbation conditions and relying on the integration of sensorimotor, visuospatial skills, and cognitive psychological function (41). Certain earlier studies have shown that chronic pain can alter the reaction time through affecting cognitive psychological function (42, 43) and motor sensory integration (44–46). As an example, one study measured hand and foot reaction time in response to light stimulation among individuals with and without chronic neck pain, the results showed that people with chronic neck pain had slower hand and foot reactions along with poorer hand-eye coordination (47). In addition, another study investigated the impact of chronic musculoskeletal pain on simple and choice foot reaction time in the elderly, indicated that both pain and light interferences contributed to slower foot reaction time, which hindered physical response and increased vulnerability to falls (39). Furthermore, in relation to trunk reaction time during slip perturbation, a study manifested no significant difference between individuals afflicted with cLBP and those without such pain (48).

Inconsistent with the above research, a study measured the ankle muscle reaction time after inducing slip perturbations on a treadmill, and found that compared with healthy individuals, cLBP exhibited faster reaction time on the dominant tibialis anterior muscles. This suggests that cLBP patients may adopt a dominance-dependent compensatory strategy to enhance dynamic balance control (49). Additionally, Tomasz Sipko et al. speculated that cLBP patients exhibited shorter preparation time in the sit-to-stand task due to adopting posture compensation strategy (50). Furthermore, a comprehensive systematic review and meta-analysis draw the conclusion that chronic pain patients, including those with fibromyalgia, had faster reaction time toward pain-related information compared to asymptomatic individuals (51). In view of the above research, it's reasonable to assume that chronic pain might play an important role in response to sudden postural perturbation, the discrepancy among this research could be attributed to gender and task differences. In the present study, we compared the limb movement responses to sudden postural perturbation in individuals with and without cLBP and found that cLBP had shorter reaction time, which aligns with the findings of prior studies by Sung PS, et al. (49) and Sipko T, et al. (50), inferring that pain might contribute to hypervigilance or facilitated attention (51). Individuals with cLBP may exhibit heightened attentional focus toward potential threats to the spine, leading to faster motor responses in a postural perturbation paradigm. We still observed that the shortened reaction time mainly occurred during PMT phase, indicating that this alteration might primarily be influenced by the central components of the neuromuscular response process, specifically the cognitive and psychological dimensions. Moreover, during the cognitive interference task, participants maintained faster response times under high-attention conditions, even when pain was present (52). Evidence from an EEG study indicates that visual task response times are inconsistent under painful conditions, this suggests that in a state of chronic pain-related hyperarousal, maladaptive alterations in attention are potentially associated with abnormal changes in neuronal gamma oscillations (53).

The relationship between chronic pain and reaction time is complex and not yet fully understood, given the involvement of cognitive, psychological, and peripheral musculoskeletal factors. One possible interpretation of our results is that the reduction in reaction time in cLBP patients reflects a maladaptive compensatory mechanism, rather than a functionally beneficial adaptation.

### Muscle fatigue and reaction time

Patients with cLBP are more prone to experience exercise-induced muscle fatigue (9). Meanwhile, some previous studies have highlighted that exercise-induced muscle fatigue might serve as a significant factor to alter the postural control as well as prompt the occurrence or recurrence of low back pain. For example, one study performed by Allison GT et al. using bilateral arm raising test to conduct postural perturbation, found that the anticipatory response of external oblique muscle would be facilitated after trunk extensor muscle fatigue, and declared that the activation latency change of the trunk postural muscles caused by fatigue may be related to the occurrence of low back pain (54).

Furthermore, by inducing abdominal muscle fatigue, Allison GT et al. also found that the magnitude of muscle activity during anticipatory postural adjustments was decreased about 20% in both rectus abdominis (fatigued muscle) and erector spinae (not fatigued), and they implied this muscle activity might rely on central fatigue effect (55). A study comparing muscle activation patterns following postural perturbation before and after fatigue, showed that patients with cLBP exhibited temporal characteristics of muscle activity that, after fatigue, closely resembled those of healthy controls. This convergence may reflect a compensatory mechanism rather than a true normalization of neuromuscular function (56). In our study, we observed the TRT of cLBP were significantly prolonged after local lumbar back muscle fatigue, especially in PMT phrase. These observations indicated that 1) muscle fatigue caused a negative impact on cLBP to cope with sudden postural perturbation; 2) PMT seemed to carry more weight in the whole reaction time. We have reason to deem that fatigue is not an independent or limited factor in the central and peripheral system, and the relationship is intricate and mutually influential, aligning with the viewpoint of Morris SL, et al.'s research (55). Voluntary activation typically declines during maximal voluntary isometric contractions, reflecting the development of central fatigue, which is accompanied by reduced motor unit firing rates (57). The fatigue effect produced by the BST may be more centrally mediated. Decreased attentional capacity and cognitive performance represent typical manifestations of central fatigue (58). Therefore, the observed prolongation of reaction time may reflect muscle fatigue-related changes in cognitive and psychological processes (59, 60).

Interestingly, the pre-muscle fatigue TRT, PMT and EMD were not significantly different from post-muscle fatigue in HC group. We considered that this could be attributed to the ample neural resource capacity inherent in healthy individuals (61). Even under conditions of fatigue, they possess adequate neural resources to effectively address abrupt perturbations, negating the need for alterations in reaction time, as also described by Le Mansec Y et al. (5). Additionally, we also noted that both the pre- and post-muscle fatigue EMD remained unaltered in individuals with and without cLBP. Notably, some previous similar studies have yielded different results. For example, Sajjad Abdollah et al.'s (20) study revealed that the EMD of lower limb muscles (gastrocnemius, tibialis anterior, and semitendinosus) in basketball players with cLBP had changed through inducing lower limb muscle fatigue. Furthermore, another study performed intermittent isometric knee flexions at 60% of the maximum voluntary contraction until failure, and demonstrated that fatigue training resulted in shorter EMD of knee muscles (biceps femoris, semitendinosus, etc.) in healthy individuals with higher training status (62). The reason for the discrepancy between our research and aforementioned study might be differences in fatigue loading mode

(trunk fatigue vs. limb muscle fatigue), tested muscle location (deltoid vs. lower limb muscles), participant training status, and reaction time task types are all important reasons for inconsistent EMD results. Specifically, the target muscles in the cited studies were subjected to fatigue load, resulting in altered EMD. Conversely, the deltoid muscle was not undergoing fatigue load in our study, likely contributing to the stability of EMD. In other words, EMD is the time interval between muscle electrical signal excitation and muscle torque signal generation, and is more closely related to local tissue metabolic changes (63), while the deltoid muscle was unaffected by fatigue load in our research, which might be the reason why EMD remained stable.

### Pain related fear-avoidance and reaction time

The patients with cLBP often accompany a certain degree of pain-related fear beliefs, and the severity of these beliefs might be associated with reaction time delay. As an illustration, one previous study, executing a seated rapid arm flexion in self-initiated and cued conditions, found that the delay of postural adjustments time was correlated with fear-avoidance beliefs in cLBP (64). Similarly, another study focusing on reaction time under dual-task conditions (performing an auditory task while receiving electrical stimulation) demonstrated that the reaction time was prolonged more obviously in cLBP, compared to asymptomatic individuals. Moreover, regression analyses unveiled that high pain-related fear was associated with reaction time delay (65).

As previously stated, the change of reaction time was correlated with pain-related fear beliefs as well as ascribed by muscle fatigue effect. However, it has not been clearly reported whether the changes of reaction time induced by muscle fatigue are related to pain-related fear beliefs. In this study, through observing changes of reaction time before and after muscle fatigue in cLBP and performing correlation analysis with pain and fear-avoidance beliefs, we identified a positive correlation between MD_TRT_ and MD_PMT_ with VAS and FABQ separately. The results suggest that pain and fear-avoidance beliefs might serve as cognitive and psychological mechanism for reaction time delay during the fatigue process. Fatigue-induced neuropsychology alterations represent a clinically relevant factor in the management of patients with cLBP. Besides, BST-TET exhibited no significant differences between cLBP and healthy individuals, and also no correlation with VAS and FABQ. We attributed this phenomenon to the fact that participants in our study were relatively young and often possessed better self-efficacy. They were willing to regard the difficult tasks as a challenge rather a danger to avoid, which is in echo with the perspectives of Vincent HK et al.'s study (66).

### Limitations

This study still has certain limitations. Firstly, the need for immediate dynamic disturbance testing after fatigue induction, coupled with current technical constraints, have forced us to rely on subjective fatigue assessment. Therefore, integrating sEMG (median frequency analysis) to objectively quantify BST fatigue levels is therefore a key methodological advancement planned for future work. Secondly, we didn't investigate the muscle fatigue effects of BST on trunk muscles, such as rectus abdominis, oblique muscles, and transversus abdominis, etc. Future research should aim to explore the response time on trunk muscles, which will contribute to a better understanding the potential mechanism of reaction time alteration induced by muscle fatigue. Thirdly, the lack of central function assessment of reaction time in our research could be addressed by incorporating brain function devices, such as electroencephalography (EEG) and functional near-infrared spectroscopy (fNIRS). This integration will further enhance the understanding of neuromuscular processes. Lastly, we only observed simple reaction time and didn't investigate other types of reaction time such as discrimination reaction time or choice reaction time. Future study would focus on reaction time mentioned above from the perspective of cognitive load, which might be conductive to comprehend the relationship between muscle fatigue and reaction time.

## Conclusions

This study measured the reaction time under postural perturbation conditions before and after exercise-induced muscle fatigue in individuals with and without cLBP, and also conducted the correlation analysis between the degree of reaction time changes and pain-related fear-avoidance beliefs. The study's results unveiled that both pain and muscle fatigue could alter the reaction time during neuromuscular control process, and these changes are dominated by cognitive-psychological PMT. Pain intensity and fear-avoidance beliefs were independently associated with prolonged TRT. These findings highlight the clinical relevance of integrating muscle fatigue monitoring and evidence-based, fear-targeted psychological interventions into the systematic rehabilitation framework for chronic low back pain.

## Data Availability

The original contributions presented in the study are included in the article/Supplementary Material, further inquiries can be directed to the corresponding authors.
